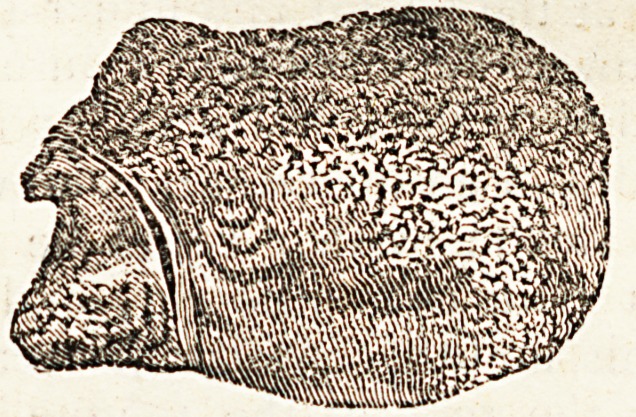# Case of Calculus Discharged by the Mouth

**Published:** 1823-06

**Authors:** 


**Art. IV.**—*Case of Calculus discharged by the Mouth.* Communicated in a Letter to the Editors, from a Constant Reader.

In presenting you with the outlines of a case of rather unusual occurrence in our profession, and at the same time of so interesting a nature, much apology seems wanted on my part for the imperfect manner in which it has been drawn up for your inspection: but, when I acquaint you that the subjoined fragments were the only information I have been able to obtain respecting its history, I trust, from its singularity, I may in some degree be justifiable in transmitting them, as they are, to your notice. Should you deem them of importance enough to lay before the public, you will permit me to intimate that they were collected from a poor, infatuated, old man, a resident, at present, in one of the Freemen's Hospitals belonging to this town.

On the 4th of September last, I attended Robert Cook, ætat. seventy-seven, a smith, for a slight contusion on the shoulder, received by slipping his foot on the stairs of the hospital. On his recovery, he showed me a calculus which he had passed from his mouth some years ago, and which I have now the honour of sending you. He is a superannuated freeman, very deaf, and extremely ignorant, having never had a knowledge of his letters or pen. The loss of such valuable acquisitions as these rendered his description of it, at first, nearly unintelligible; but, by frequent interrogations and cross-examinings, I have been able, I hope, to convey a faint, though correct, sketch of this remarkable concretion. About fifteen years ago, this man was scratching a painful spot under the tongue with his fore-finger nail, and broke the skin; when, to his great surprise, a gritty substance came out, white like ivory, rough like a shell, and shaped exactly like a plum-stone: indeed, so much so, that he thinks, if he had cracked it, he would have found a kernel in it! The sore afterwards readily healed up; yet still to this day there remains an opening, so small as scarcely to admit the point of a pin. Unfortunately, I could not obtain a sight of this calculus, as it had some time ago been swept from the shelf, and lost. About half a year after this stone came away, he found a swelling in the neck, a hard knotty swelling on the right side, just above the extremity of the right corner of the os hyoides, and nearly about the place where the submaxillary gland lies. After it reached its full size in the neck, it grew very sore far back in his mouth, at the lateral and under-part of the tongue. It continued to grow so very red and painful in the neck, that he was recommended to have it lanced by a surgeon; but he did not relish the idea of an operation, so he would not consent. He could get no sleep; neither could he swallow any thing without very great pain and suffering, and the saliva flowed continually from his mouth. At last it became so exquisitely sore and tender at the root of the tongue, that he passed his finger in one day to the affected part, to ascertain the cause. He found near its root the larger portion, or base, of the calculus protruding through the skin. He was panic struck! for he could not imagine what he could have got hold of. However, by passing in again his fore-finger to the part, while he fixed his thumb underneath his jaw as a sort of support to it, he found this hardened substance moved; and, by "*joggling*" it, as he said, repeatedly with his finger, it sprung out of its place at last with a *jerk;* the larger piece coming away first, the fundus of which was the part he felt with his finger projecting into his mouth; and afterwards the smaller piece, or root of it, or its pedicle, which he says was the part attached to the tongue. No blood or matter followed, and the cavity or cyst wherein it was contained soon got well, (though a cavity yet remains,) as also his neck, and he became able to swallow again as well as ever; feeling no more pain or uneasiness there or in any other part since. I took an opportunity of examining his mouth when I attended him for the contusion, and can pass my finger down into a sac or pouch, which he says it was embedded in, which is between the right side of the body of the tongue and the angle of the inferior maxillary bone, below where the fang of the last dens molaris or sapientiæ had been. It feels smooth in many parts, and in others rough and uneven, as if fleshy strings or cords were interwoven in it, and crossed it like what we feel with our fingers in the cavities of the heart. This concretion was of a soft, sandy texture when first discharged, and might readily have been bruised between the finger and thumb; though now, from process of time, it is harder and more compact. Its colour was the same then as it now is,—viz. of a pale brown, similar to the usual appearance of the different kinds of phosphates; and like them, also, seems to be composed of concentric laminæ of earthy matter. The constant motion to which it must long have been subject during its formation, from the muscles of mastication, deglutition, and voice, has probably been the occasion of that smooth polished surface where the apparent juncture of the larger portion to the smaller is seen, so as to form a socket or joint-like appearance. Its general aspect is rough, porous, or spongy-like, or somewhat resembles the cancelli of the caput femoris when sawn through. Its weight is two drachms forty-eight grains.

I am sorry I am not able to lay before the readers of this miscellany a more faithful detail of the concomitant minutiæ regarding the formation of this calculus. Were I to attempt to do so, I would be deviating from my notes, and entering into theoretical speculations of my own,—a purpose quite foreign to the object of this communication. I have, therefore, stated nothing but what hath fallen from the patient's own lips, leaving gentlemen to form their own conclusive opinions after the case has once been divulged to them.

*Newcastle-upon-Tyne; April 16th, 1823.*

## Figures and Tables

**Figure f1:**